# Rotationplasty for Severe Congenital Femoral Deficiency

**DOI:** 10.3390/children8060462

**Published:** 2021-06-01

**Authors:** Corey B. Fuller, Craig H. Lichtblau, Dror Paley

**Affiliations:** 1 Department of Orthopedic Surgery, Loma Linda University, Loma Linda, CA 92354, USA; CoreyFullerMD@gmail.com; 2 Paley Orthopedic and Spine Institute, St. Mary’s Hospital, West Palm Beach, FL 33407, USA; C.Lichtblau@chlmd.com

**Keywords:** rotationplasty, congenital femoral deficiency, deformity

## Abstract

Rotationplasty is a reconstructive option for severe congenital femoral deficiency (CFD). The senior author (D.P.) developed five new rotationplasty techniques for use in CFD based on the Paley classification, including the Paley–Brown (fusion femur to pelvis), Paley (fusion femur to femoral head), Paley–Winkelman (insertion tibial condyle to acetabulum), PaleySUPERhip–Van Nes (hip osteotomy with knee fusion) and PaleySling–Van Nes (hip reconstruction with knee fusion revision) rotationplasty techniques. The purpose of this study is to retrospectively evaluate the complications, radiographic outcomes and need for secondary surgery in 19 rotationplasty cases performed by the senior author (D.P.) for severe CFD from 2009 to 2019. Rotationplasty comprised only 2% of the authors treated CFD cases during this period. Average age at surgery was 8.6 years old. Average follow-up was 3.3 years. Sixteen concomitant procedures were performed including temporary arthrodesis, tibial osteotomy and SUPERhip procedure. The most common complication was wound necrosis/dehiscence, which occurred in 52% of the cases related to the circumferential incision and required a total of 31 additional debridements. Additional complications were successfully treated and included sciatic nerve palsy decompressed by abducting the femur, a tibial delayed union that underwent bone grafting, two distal femur failed epiphysiodesis treated by revision with one osteotomy and a thigh compartment syndrome requiring debridement. Indication specific rotationplasty successfully addresses the severe degree of femoral deficiency, deformity, and discrepancy in patients with CFD, despite high rates of wound complications.

## 1. Introduction

Congenital femoral deficiency (CFD) is a rare congenital disorder with a reported incidence of 1:50,000 births [1,2,3]. It presents with a wide spectrum of severity from mild femoral hypoplasia to severe femoral deficiency with deformity and length discrepancy. The natural history of CFD is a progressive limb length difference, but the deformities and contractures persist but do not progress [4]. Surgical management of CFD can be challenging and options include reconstruction with lengthening with reconstructable hip and knee joints versus amputation or rotationplasty with prosthesis for more severe cases [5,6,7,8].

Rotationplasty (RP) is an old concept that has been used for congenital and acquired lower extremity bone loss. It is a limb-sparing procedure that rotates the involved extremity 180 degrees so that the ankle functions like a knee joint and can be fitted with a below-knee type prosthesis onto the foot that functions as the tibia. Rotationplasty is a well-recognized reconstructive option of treatment for congenital femoral deficiency (CFD) and limb salvage for lower extremity sarcomas [9,10,11,12,13,14].

Rotationplasty was originally described for treatment of a patient with bone loss from tuberculosis in 1927 [15] and was popularized by Van Nes for treatment of CFD in 1950 [16]. This type of rotationplasty also converts the ankle to a knee, while the original knee is fused straight, and the hip is left free floating relative to the pelvis. Krajbich popularized this method for use in CFD and performed it through a long lateral S-shaped incision [17]. Since the proximal end was not anchored, it had a tendency to derotate, undoing the benefit of the procedure. Brown described a new type of rotationplasty (first presented in Dallas in 1996 at a meeting on CFD, then published in 1998 as a book chapter in an AAOS monograph from that meeting [18], finally publishing his case series in 2001 [19]) by using a circumferential incision and fusing the distal femoral remnant to the side of the ilium, thus also converting the knee joint to a stable hinge like hip. Anchoring the femur to the pelvis eliminated the problem of the leg derotating. The lower limb was frequently fused to the ilium in valgus due to the inclination of the lateral ilium. It also protruded laterally a lot since the new hip joint was laterally located compared to the normal hip joint. 

**Figure 1:**
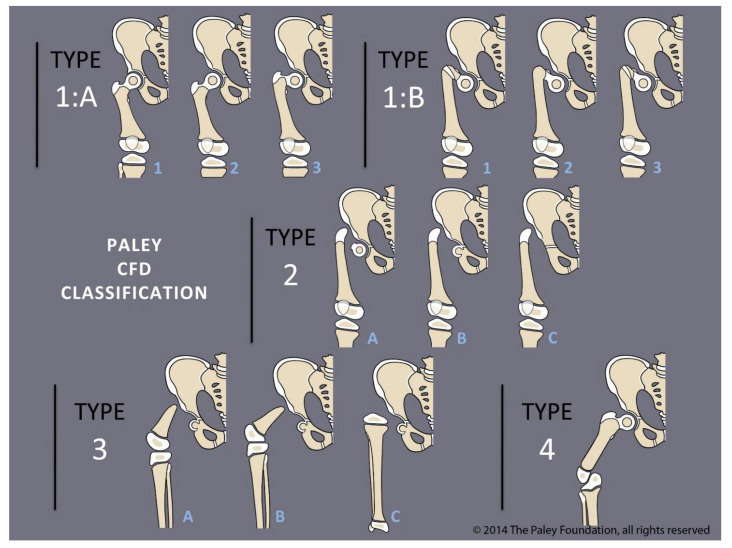
Paley CFD classification.

**Figure 2:**
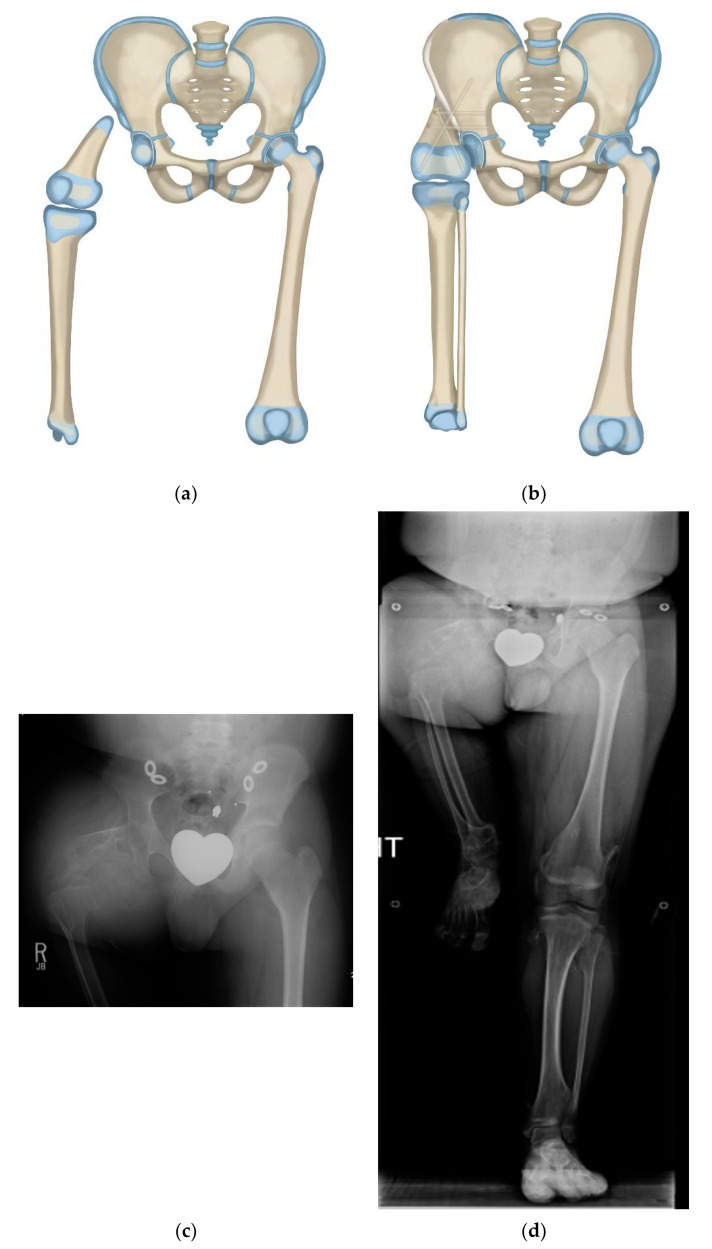

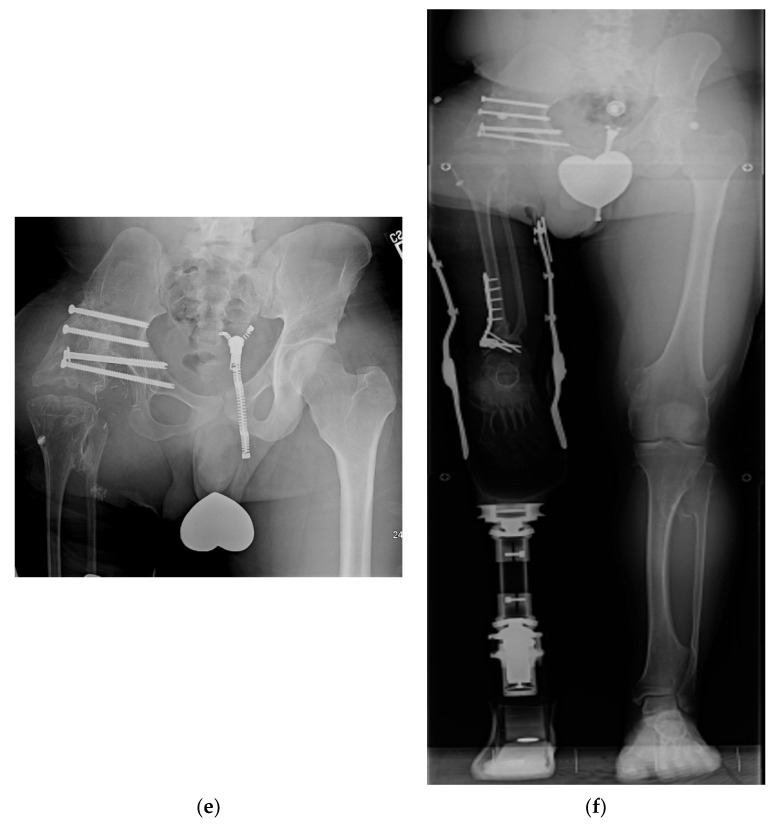
(**a**) Illustration of CFD Paley type 3 (**a** or **b**), (**b**) Illustration after Brown rotationplasty for Paley type 3 (**a** or **b**), (**c**) AP pelvis x-ray of 14-year-old boy with Paley type 3b CFD, (**d**) Preop standing erect leg x-ray of same boy, showing that the ankle is at the level of the opposite knee (incidentally this patient also has multiple osteochondromas), (**e**) AP pelvis radiograph in same boy, 7 years after healed Brown rotationplasty, (**f**) Sanding radiograph with prosthetic 7 years after Brown rotationplasty with supramalleolar osteotomy for ankle realignment. Clinically he has excellent gait and function.

**Figure 3:**
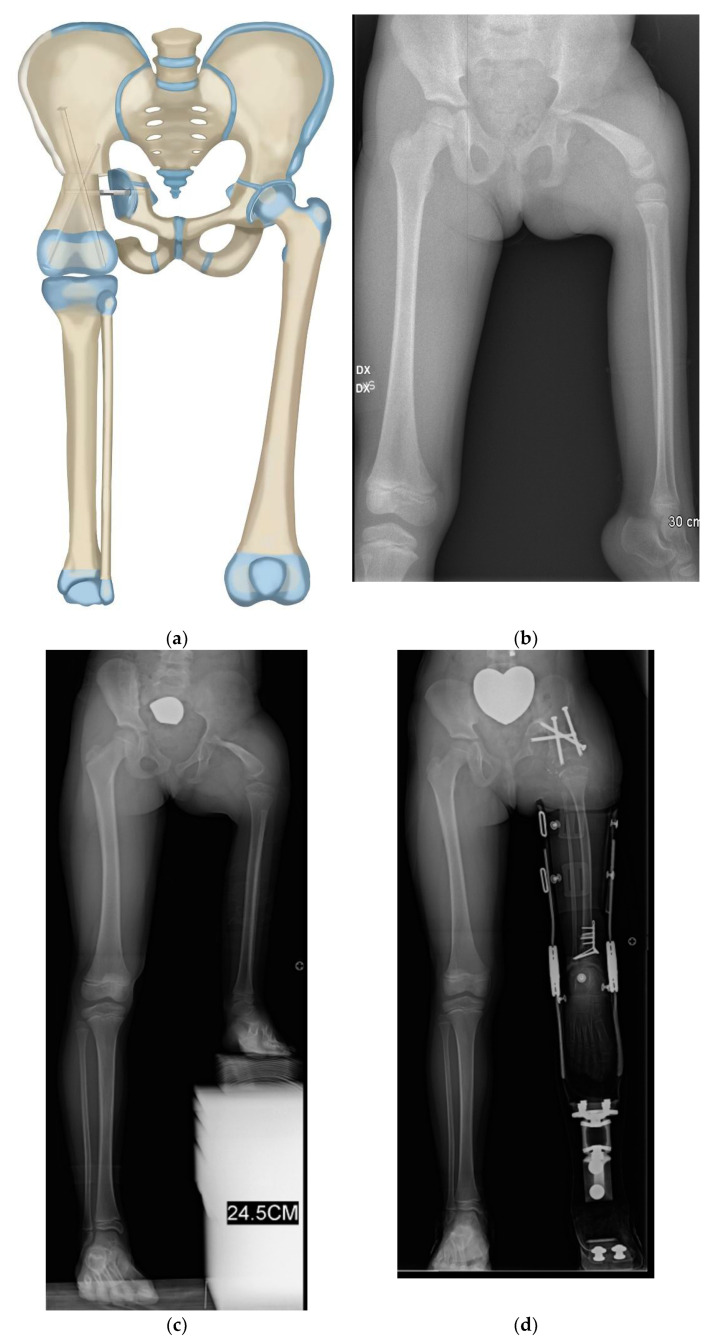
(**a**) Paley–Brown Rotationplasty illustration for Paley type 3 (**a** or **b**), (**b**) AP pelvis radiograph in 3-year-old girl with Paley type 3a CFD, (**c**) Standing long radiograph showing the left ankle is at the level of the right knee, (**d**) Standing long radiograph two years after Paley–Brown rotationplasty. The ankle is at the level of the opposite distal femoral physis (level of knee center of rotation). Clinically she has excellent gait and function.

Paley modified the Brown technique in 1997 by fusing the distal femoral remnant to a Chiari pelvic osteotomy (Paley–Brown technique) instead of the lateral ilium. The advantage of fusing to a Chiari osteotomy instead of the side of the pelvis is the medialization of the hip joint to improve hip mechanics and cosmesis. The Paley–Brown modification also makes it easier to achieve the correct frontal plane alignment. The fusion of cancellous bone of the distal femoral remnant with cancellous bone of the ilium at the Chiari osteotomy in an axially loaded transverse osteosynthesis line also increases fusion potential compared to the vertically sheared Brown fusion line. Paley types 2 and 3 CFD (Figure 1) are the best indications for the Brown (Figure 2a–f) and Paley–Brown (Figure 3a–d) rotationplasties. 

**Figure 4:**
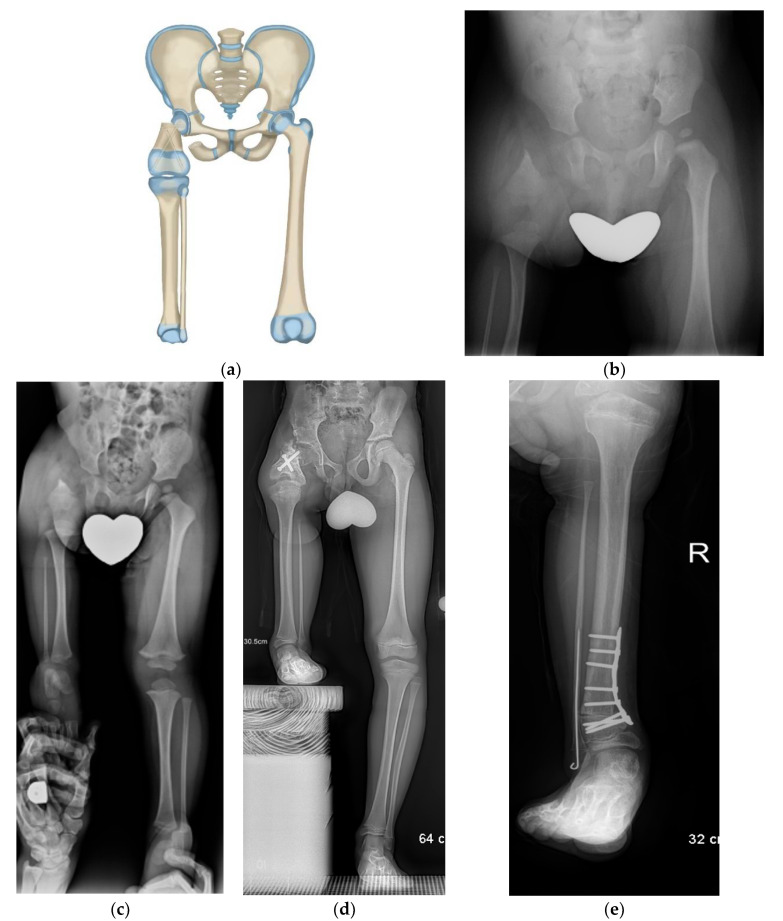
(**a**) Paley rotationplasty illustration. (**b**) AP pelvis of 2-year-old boy with CFD Paley type 3a. (**c**) Standing radiographs in same boy before surgery showing ankle is at level of opposite knee. (**d**) Since the femoral head was mobile a Paley rotationplasty was performed. Standing long radiograph in same boy, 8 years after Paley rotationplasty. (**e**) To improve his prosthetic fitting he had a varus derotation surpramalleolar osteotomy performed 8 years after the original rotationplasty. Clinically he is very sports active and has excellent gait and function.

In a subset of Paley type 3a or 3b or in Paley type 2a CFD patients, there is a mobile femoral head in the acetabulum. This can be determined before surgery using MRI. In these cases, the distal femoral remnant can be fused directly to the femoral head (Paley rotationplasty) (Figure 4a–e). Paley started using this method for selective cases since 2012. This creates a more 3-dimensional hip motion flexing and extending through the rotated knee joint and abducting-adducting and axially rotating through the femuro-acetabular joint.

**Figure 5:**
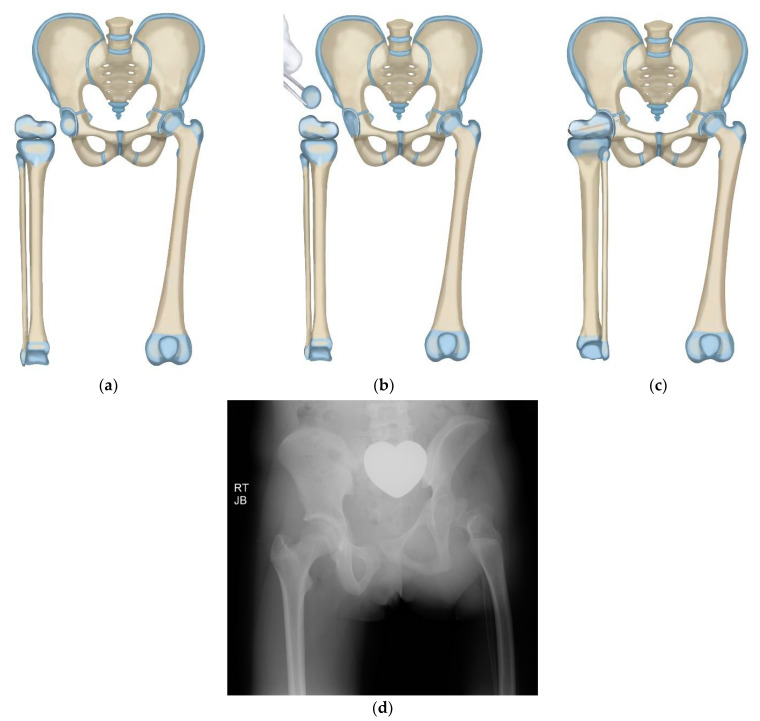

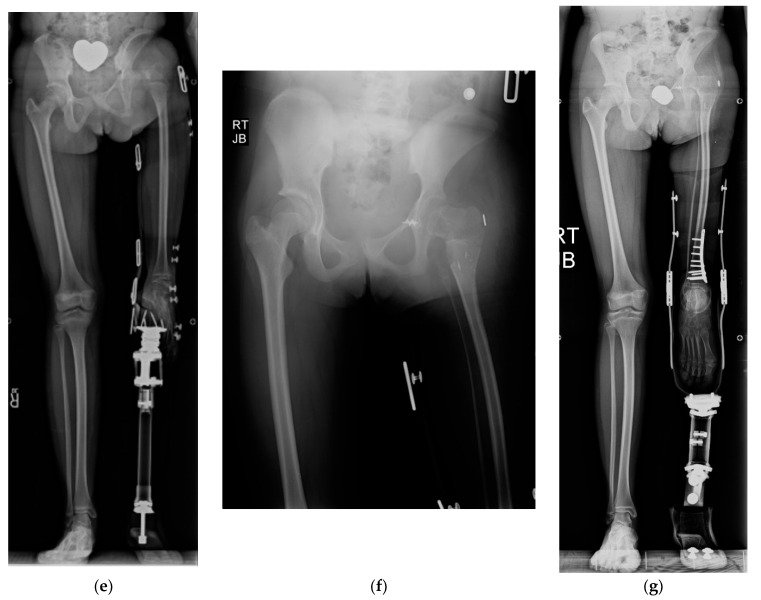
(**a**) Illustration of CFD Paley type 3c. There is an ankylosed knee with a small distal femoral remnant. (**b**) Illustration showing the femoral head is enucleated to make room for the femoral condyle or tibial plateau in the acetabulum. (**c**) Paley–Winkelmann rotationplasty illustration, inserting the femoral condyle remnant into the acetabulum secured with a hip tethering suture. (**d**) AP pelvis radiograph of a 12-year-old girl with CFD Paley type 3c. (**e**) Standing long radiograph of same girl showing the ankle is at the level of the opposite knee. (**f**) AP pelvis radiograph of same girl after Paley–Winkelmann rotationplasty with femoral condyle in the acetabulum. The tethering suture anchor is seen. (**g**) Standing radiograph of same girl 5 years after Paley–Winkelmann rotationplasty. She has excellent function of the new hip joint and can walk and run with very minimal limp.

Neither the Brown, Paley–Brown, nor Paley types of RP are indicated if there is complete absence or ankylosis of the knee joint with an absent femur (Paley type 3c). Winkelmann articulated the lateral plateau of the tibia with the acetabulum when performing rotationplasty for total femur resection in sarcoma patients. Paley modified the Winkelman RP in 2014 to treat CFD type 3c (Figure 5a–g). He used the same circumferential incision as in the Paley–Brown without the second circumferential incision used in the Winkelmann to shorten the thigh since the limb is already shortened, adding a suture tethering system to maintain the tibia in the hip joint (e.g., syndesmotic suture washer device or suture anchor device). With this method the lateral side of the tibial plateau or dysplastic remnant of distal femur articulate with the acetabulum after first enucleating the femoral head (Paley–Winkelmann rotationplasty).

**Figure 6:**
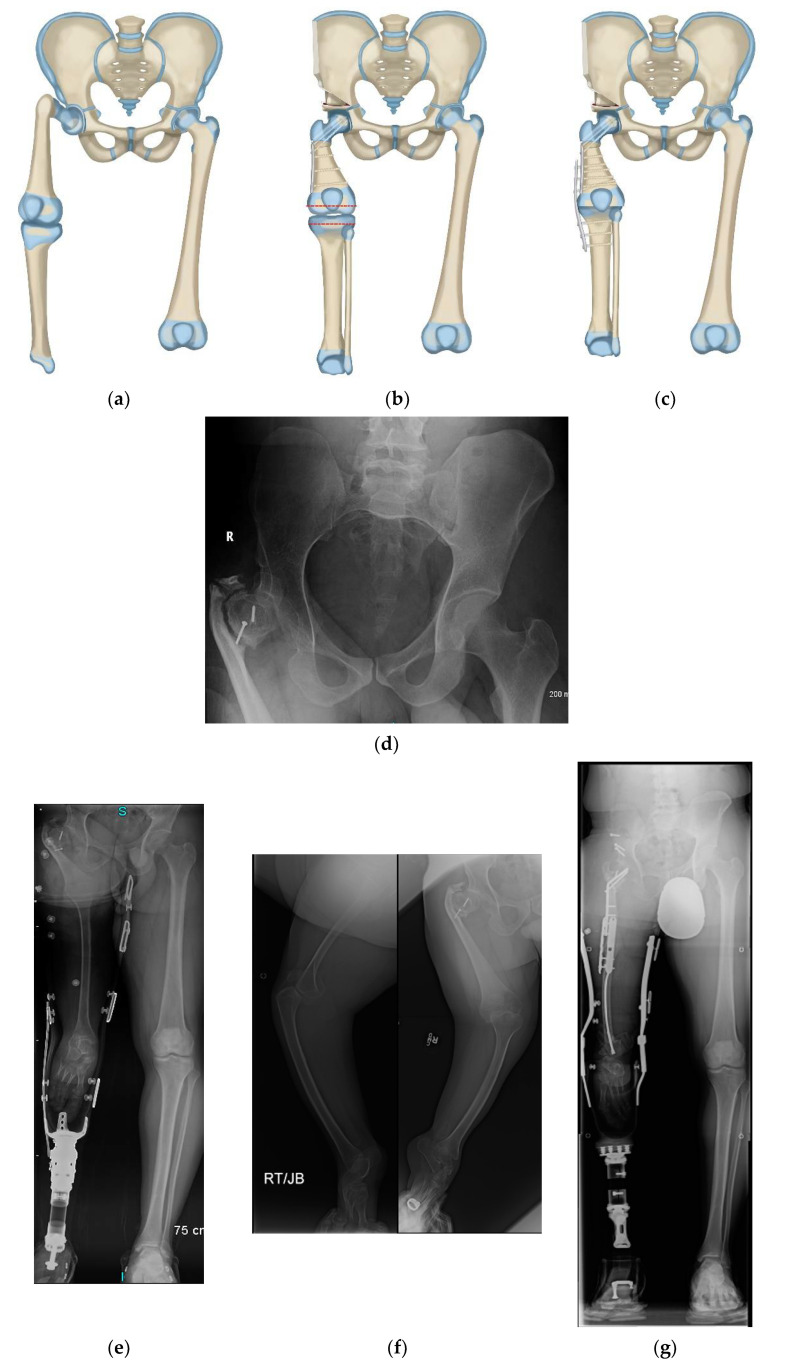
(**a**) Illustration of CFD type 1a3 or 1b. (**b**) Illustration showing the first step which is SUPERhip procedure with resection of knee joint. (**c**) Illustration showing the second step which is PaleySUPERhip–Van Nes rotationplasty at level of the knee fusion. (**d**) AP pelvis on 36-year-old man with CFD type 1b with prior failed hip surgery. (**e**) Standing radiograph in same man showing ankle at level of opposite knee joint. (**f**) Long lateral and AP radiographs showing the knee joint in the same patient was unstable, deformed and subluxated. (**g**) Standing radiograph one year after PaleySUPERhip–Van Nes procedure including SUPERhip, knee fusion and supramalleolar osteotomy, wearing rotationplasty prosthetic. Ankle is at level of opposite knee. This procedure greatly improved his quality and of life, gait and function.

**Figure 7:**
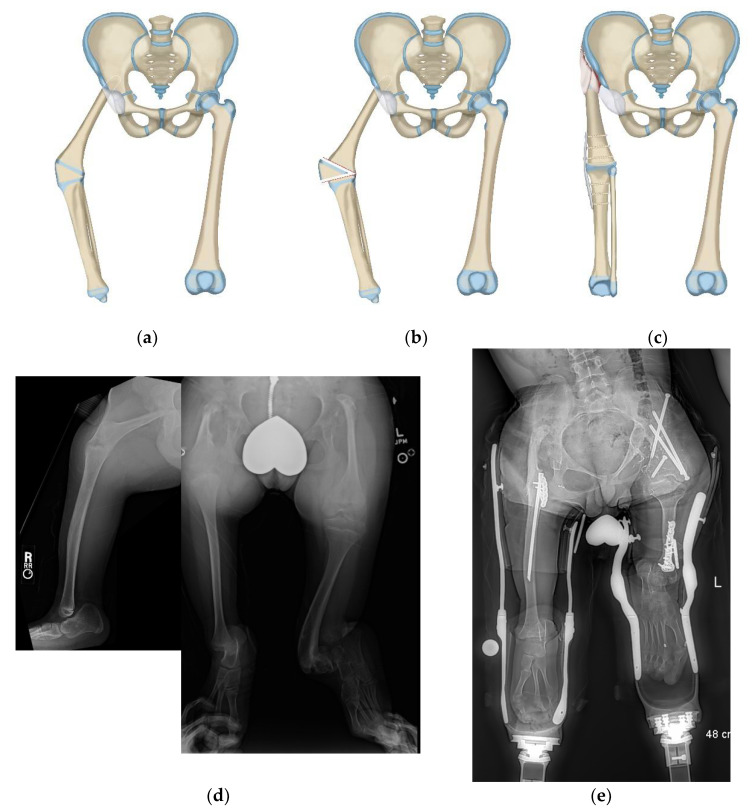
(**a**) Illustration of CFD type 2c with congenital knee fusion with knee flexion deformity. The fibrous femoral neck anlage tethers the upper femur from migrating proximally. (**b**) Illustration showing the first step which is correction of knee flexion deformity with excision of distal femoral physis. (**c**) Illustration of the second step which is PaleySling–Van Nes rotationplasty. The rotationplasty is performed through the knee fusion site. The proximal femur is stabilized using the fascia lata by creating a sling around the proximal femur. The sling plus the fibrous neck anlage stabilize the upper femur from migrating proximally and while preserving the hip flexion-extension mobility (**d**) AP long radiograph (right) of a 14-year-old boy with bilateral CFD. The right side is classified as type 2c and also has a congenital knee fusion as seen on the long lateral radiograph (left). On the left side it is classified as a CFD type 3b and the knee joint is present and functional. (**e**) Standing long radiograph one year after bilateral rotationplasty performed in two separate surgeries. On the right side the rotationplasty was performed through the congenital knee fusion site together with a sling procedure at the hip (PaleySling–Van Nes). On the left side a Paley–Brown rotationplasty was performed together with a supramalleolar osteotomy for realignment.

Finally, rotationplasty may be indicated in CFD cases that have a severely deformed proximal femur typical of Paley type 1a_3_ or 1b as well as in patients with CFD associated congenital knee fusion (these can be Paley types 1, 2 or 3). These patients can be treated with a long S shaped incision with a modified Van Nes rotationplasty including knee fusion combined with one of the following two treatments at the hip joint: (1) SUPERhip procedure to treat the severely deformed proximal femur for types 1a_3_ or 1b (PaleySUPERhip–Van Nes) (Figure 6a–g) or (2) Femoral Sling procedure for Paley type 2 or 3 CFD (PaleySling–Van Nes) (Figure 7a–e). Both prevent derotation of the hip after the rotationplasty due to stabilization of the hip by either the SUPERhip or Sling procedures. In the CFD with a congenital knee fusion type, rotationplasty is performed through the congenital knee fusion level (PaleySling–Van Nes rotationplasty). 

This purpose of this study is to retrospectively evaluate the results of these new rotationplasty methods for the treatment of CFD.

## 2. Materials and Methods

The senior author (D.P.) has performed rotationplasties for CFD since 1997. Paley performed 20 Paley–Brown rotationplasties between 1997 to 2008 at two institutions in Baltimore. These Baltimore cases represents the senior author’s learning curve. This study represents the senior author’s experience since moving to West Palm Beach, Florida at St. Mary’s Medical Center from 2009–2019. All rotationplasties performed by the senior author (D.P.) for severe CFD from 2009 to 2019 were included in this study (Table 1). The average age at surgery was 8.6 years (range 2–36 years). Each case was classified according to the Paley CFD classification [8,20]; 1 Paley type 1, 3 Paley type 2 and 15 Paley type 3. There were 10 Paley–Brown rotationplasties, 5 Paley rotationplasties, 2 Paley–Winkelmann rotationplasties, 1 Brown rotationplasty and 1 PaleySUPERhip–Van Nes rotationplasty. There were no PaleySling–Van Nes RPs in this series but two have been performed by the author in the past two years since this study. They are included in the discussion but not in the results. In addition to the index rotationplasty procedure, there were 16 planned concomitant procedures performed; 8 application of temporary hip/knee spanning using internal or external fixation, 7 tibial osteotomies to correct rotation or valgus deformity secondary to associated fibular hemimelia, and 1 SUPERhip procedure in a patient who had the PaleySUPERhip–Van Nes rotationplasty.

This study retrospectively reviewed complications, radiographic outcomes and additional elective surgeries during the postoperative period. All complications and any additional elective surgeries during this follow-up were also recorded. 

### 2.1. Preparatory Steps for Paley–Brown and Paley Rotationplasty Surgical Procedures

**Step 1:** Racket incision is made just proximal to the skin crease in the popliteal fossa with a longitudinal extension perpendicular to this incision that extends to the anterior superior iliac spine.

**Step 2:** Create full thickness flaps with the superficial fascia to preserve blood supply to the skin.

**Step 3:** Identify the saphenous vein medially, preserve it and dissect the saphenous vein proximally to where it joins the femoral.

**Step 4:** Dissect the femoral artery and vein from this point distally to knee. Ligate, staple or cauterize all side branches of the femoral artery and vein, including the profunda femoris and circumflex vessels. The femoral artery and vein need to end up as tubular conduits without tethering branches from the inguinal canal to where they divide at the popliteal fossa. 

**Step 5:** Dissect free the sartorius muscle and release it distally from the tibia. Take care to preserve and dissect free the saphenous nerve which lies adjacent to this muscle. Follow the saphenous nerve back to identify and protect the femoral nerve. 

**Step 6:** Release the hip adductor muscles off of the medial femur. Staple or cauterize the large number of vessels in this area.

**Step 7:** Release the remaining medial hamstrings off of the tibia.

**Step 8:** Go to the lateral side and dissect free the fascia lata. Cut across the iliotibial band at the tibia and reflect this structure proximally.

**Step 9:** Identify the posterior border of the biceps femoris muscle and then find the common peroneal nerve. 

**Step 10:** Follow the common peroneal nerve distally to where it enters the peroneal fascia and decompress it at this first tunnel. Make a transverse incision across the fascia of the lateral and anterior compartments. Find the intermuscular septum and release it to decompress the deep peroneal nerve at the second tunnel. 

**Step 11:** Now that the peroneal nerve is visible release the biceps tendon from the fibula.

**Step 12:** Follow the peroneal nerve proximally to where it is joined by the posterior tibial nerve and becomes the sciatic nerve.

**Step 13:** Reflect back the gluteus maximus and identify and release the piriformis muscle.

**Step 14:** Release the rest of the external rotators from the femur.

**Step 15:** Decompress the sciatic nerve all the way to the sciatic notch. Release the hip abductors off of the proximal end of the femur.

**Step 16:** Elevate the quadriceps muscles off of the femur extraperiosteally, starting from lateral to medial. Release the quadriceps distally either at the proximal end of the patella if it present or at the level of the knee joint if there is no patella. Lift the quadriceps from distal to proximal off of the femur.

**Step 17:** Release the rectus femoris tendon off of the anterior inferior iliac spine.

**Step 18:** Find the psoas tendon and release it from the femur and tag it so that it does not retract into the pelvis.

**Step 19:** Release the fibrous anlage of the femoral neck off of the femur.

**Step 20:** Open the superior capsule and examine if the femoral head is present, fused to the acetabulum, or mobile. In the latter case consider doing a Paley type of rotationplasty instead of the Paley–Brown. 

**Step 21:** The proximal femur is now free to rotate since it is no longer tethered by any structures originating from the pelvis. On the lateral side release the tendon of the lateral head of gastrocnemius off of the femur. 

**Step 22:** On the medial side release the tendon of the medial head of the gastrocnemius off of the femur. Dissect the vessels free of the distal femur but do not damage the branches to the two heads of the gastrocnemius. 

**Step 23:** Now rotate the femur externally until the posterior aspect is facing anterior. If there is a flexion contracture of the knee joint (present in almost all cases), perform a posterior capsulotomy of the knee joint and straighten the knee into full extension. 

### 2.2. Paley–Brown Rotationplasty

**Step 1:** For the Paley–Brown, split the apophysis. For the Paley type there is no need to split the apophysis. 

**Step 2:** Elevate the periosteum off of the medial and lateral walls of the ilium down to the sciatic notch. Laterally elevate the periosteum off the anterior aspect of the notch to reach the ischium. 

**Step 3:** Return to the adductor muscles and resect them from their origins. This requires dissection down to the pubic rami. The adductor muscles are completely resected in the Paley–Brown since they get in the way of medializing the femur. They also have no role since the new hip joint is a knee joint with no ability to abduct or adduct. Also resect the sartorius muscle taking care not to injure the femoral nerve. For the Paley type resect only some of the adductors but leave some. 

**Step 4:** Enucleate the femoral head for the Paley–Brown. 

**Step 5:** Expose the ramus of the ischium for both Paley and Paley–Brown. The biceps origin must be released from the ischium. Resect a generous portion of the ischial ramus using a saw. This allows the sciatic nerve to move medially into this space and avoid becoming entrapped by the femoral remnant when it is fused to the ilium or the femoral head. This step is essential in the Paley–Brown and is also preferred for the Paley type too. It is not necessary for the Brown type since the femur stays lateral. 

**Step 6:** Insert a frontal plane guide wire parallel to the line going across the tops of the iliac crests. Make the Chiari osteotomy parallel to this line using a saw. 

**Step 7:** Displace the acetabular segment medially to hook onto the inside of the ilium. 

**Step 8:** Insert a guide wire into the femur perpendicular to the long axis of the tibia. Cut the femur along this wire. Depending on the length of the distal femur remnant, shorten the femur sufficiently to keep the knee joint as proximal as possible so that it is as close as possible to the anatomic hip joint level. 

**Step 9:** With the femur rotated 180°, fix the distal femur with guide wires: one retrograde from the medial femoral condyle (lateral side) and one antegrade from inside the pelvis into the lateral femoral condyle (medial side). Make sure both of these cross the distal femoral growth plate to create an epiphysiodeses (this prevents the new hip joint from growing away from the pelvis). Add a third transverse wire to fix to the acetabulum proximal to the triradiate. 

**Step 10:** Drill over each guide wire and insert three 5.5mm fully threaded screws.

### 2.3. Paley Rotationplasty

**Step 1:** Make an inferior capsulotomy and expose the femoral head in the acetabulum.

**Step 2:** Using a scalpel remove cartilage in the inferolateral aspect of the femoral head until the ossific nucleus is identified.

**Step 3:** Rotate the femur 180°. Now 45° bevel the lateral proximal corner of the femur.

**Step 4:** Insert one guide wire transversely across the femur into the femoral head. Insert a second guide wire in a retrograde oblique angle from the medial femoral condyle (lateral side) to the femoral head. Replace both of these guide wires with 5.5 mm cannulated screws. Add a third guide wire antegrade from the medial proximal femur (lateral side) to cross the lateral side of the physis into the lateral femoral condyle (medial side). The two oblique cross screws create a distal femoral epiphysiodesis.

**Step 5:** Add a temporary arthrodesis spanning plate to neutralize the forces on the hip joint.

### 2.4. Muscle Transfers and Closure for Both Paley and Paley–Brown

**Step 1:** Suture the psoas tendon to the medial head of gastrocnemius tendon (lateral side). The lateral head of gastrocnemius is left free to avoid any pressure on the femoral vessels that cross it.

**Step 2:** Flex the knee joint (new hip joint) and then advance the gluteus maximus and fascia lata-iliotibial band, to the patella if present or to the remnant of the distal quadriceps. This repair is critical to create good active extension of the hip. Make sure to leave the distal quadriceps attachment long to allow the gluteus maximus to reach.

**Step 3:** With the knee in flexion, suture the biceps muscle to the medial anterior tibia (posterolateral) next to the gluteus maximus insertion.

**Step 4:** Suture the medial hamstrings (semimembranosus and semitendinosus) to the lateral anterior aspect of the tibia (posteromedial). The gracilis and sartorius are not transferred since they were resected. 

**Step 5:** Suture the quadriceps to the fascia of the gastrocnemius muscles. Reattach the quadriceps proximally to the anterior superior spine. Since the quadriceps is very copious, resect the vastus medialis to reduce its volume. The quadriceps covers the femoral vessels. The saphenous vein and nerve also run deep to the quadriceps to reduce tension on them.

**Step 6:** In the Paley type, transfer the hip abductor muscles to the femur laterally. In the Paley–Brown there is no need to transfer these muscles.

**Step 7:** Similarly in the Paley type transfer any adductors that were preserved to the lateral femur (medial side). Care should be taken not to entrap the peroneal nerve that passes medial to the femur in the limited space between the femur and the pelvis. 

**Step 8:** Place two drains running in different directions around the femur. 

**Step 9:** A multiplanar closure is done to close the dead space. Skin edges are resected prior to closure, including to allow for the shortening and to facilitate closure. Most recently the Pinsky incision and closure method has been used (ala Dr. Mark Pinsky). The authors preference is to have a plastic surgeon close the incision. This serves several purposes. It allows for a meticulous closure to be done by a surgeon who is fresh and not tired from the long rotationplasty surgery and also is a surgeon who specializes in closure of skin flaps. 

Supramalleolar osteotomy or SHORDT

In the Paley–Brown or Brown the distal femur is fixed to the pelvis. If there is any malrotation of the ankle joint, then the ankle which serves as the new knee will be maloriented for prosthetic fitting. Furthermore, there can be ankle valgus present which should be aligned for the same reason. When there is no fibula or when the fibula is present but is at station (distal fibular physis at level of tibial plafond) a supramalleolar osteotomy can be performed from the lateral side (medial tibia) for rotation and valgus correction. If there is also some equinus the osteotomy is shortened to relax the Achilles tendon. The Achilles is never lengthened to avoid weakening the new quadriceps of the new knee. If the fibula is present but is hypoplastic so it proximal to station, the SHORDT procedure is performed (Shortening Osteotomy Realignment Distal Tibia).

Patients are monitored for circulation in the ICU. Removeable spica cast is used for 6 weeks with the hip in mild flexion and neutral coronal alignment at the limb. Physical therapy for passive range of motion of the hip and knee (original knee and ankle) are started. Patient is kept non-weight bearing 12 weeks until evidence of radiographic union. Patients are fitted with a rotationplasty prosthesis, 12 weeks after the Paley–Brown rotationplasty. In the Paley rotationplasty they are taken to the operating room to remove the temporary spanning (arthrodesis) plate or external fixator. After that they were fitted for a prosthetic device. After they received their prosthetic, gait training with the prosthetic begins and lasts for several months.

## 3. Results

Postoperative follow-up average was 3.3 years. All patients were fitted with a prosthetic device and underwent rehabilitation. All patients learned to walk with the prosthetic device and were able to walk and run at follow-up. All patients were functioning well with their prosthetic device. Seven patients underwent planned second stage removal of temporary spanning hardware crossing the hip or knee. 

Complications in the postoperative period occurred in 63% (12/19) patients (Table 2). The most common complication was flap necrosis with wound dehiscence, occurring in 52% (10/19) patients. Of these 10 patients with wound complications, 9 required additional surgery for debridement and secondary closure. Several were also treated with a vacuum assisted closure device (VAC). One patient had a compartment syndrome of his quadriceps and hamstring muscles together with skin flap necrosis. He required 15 additional surgical debridements and a VAC to achieve closure. There were 37 unplanned surgeries performed to treat complications, of which 31 were debridement for wound complications. Additional complications requiring surgery included postoperative sciatic nerve palsy that resolved with revising femoral position (abducting the leg in a Paley type due to sciatic nerve compression in the more adducted position), delayed union of a proximal tibial osteotomy that required compression and bone grafting, and two incomplete distal femur epiphysiodesis requiring further surgery. 

During the late follow-up period, 11 elective surgeries were performed. These elective surgeries included 5 osteotomies around the knee to correct coronal plane knee deformities, 3 supramalleolar osteotomies of the distal tibia, including one shortening osteotomy realignment distal tibia (SHORDT) procedure, and 1 advancement of gluteus maximus and hamstrings. The remaining two were hardware removals (Table 3).

There were 9 supramalleolar osteotomies of which 5 were the SHORDT type. Six were performed at the index procedure and 3 were performed in the late-postoperative setting (Table 3).

Postoperative pelvo-lower extremity alignment was measured in the coronal plane on the 1st postoperative radiograph taken (usually 6 weeks) and as well as radiograph done at most recent postoperative visit (average 3.3 years postop). This was measured on an AP radiograph by drawing a line across the top of the bilateral iliac crests and then a second line through the center of the knee and center of the ankle. Then the medial angle between these two lines were measured, hence 90 degree would represent orthogonal lower extremity to the pelvis, which was the goal at the time of surgery. Overall average alignment on the 1st postoperative radiograph was 98 degrees (range 86–117) and on final postoperative radiographs available, average alignment was 96 degrees (slight abduction). There were five patients who underwent osteotomies around the knee for residual or recurrent deformity in the coronal plane. In these five patients, average alignment on the 1st postoperative radiograph was 102 degrees and on final postoperative radiographs after osteotomy, average alignment was 94 degrees.

## 4. Discussion

Severe congenital femoral deficiency remains a challenging disease for which there is no easy solution. Rotationplasty is an old concept that has increasingly been used as a reconstructive option in severe CFD. Although previous rotationplasty techniques were successful in reconstructing the limb in severe CFD, they had known limitations. The senior author (D.P.) developed five different rotationplasty techniques that are modifications/advances to previous techniques. This study specifically looks retrospectively at the senior authors experience with rotationplasty in CFD and represents the largest reported series of rotationplasty for CFD.

The most common complication in this series was wound dehiscence. More than half (10/19) of the patients in this series had wound complications with flap necrosis and all of them except one required additional surgery to address this. There were a total of 31 additional surgeries required in this series, related to wound complications. Because of the exposure necessary to safely perform a rotationplasty, the skin incision required is circumferential with elevation of large skin flaps, which places the distal edges of the skin flaps at higher risk of ischemia. It is important to elevate the superficial fascia of the thigh along with the skin flaps to preserve their blood supply to reduce this risk, however it is still occurred in more than half the patients in this study. Skin flap necrosis is not unique to this series and is a well-documented complication after rotationplasty [13,14,19,21]. Brown [19] reported a 66% complication rate of flap necrosis, which although his series is relatively small, is consistent with our experience in this study. Although this complication appears to be common, it is also very manageable and all patients in this study at final follow-up had healed wounds without long term sequalae. 

One patient developed significant muscle necrosis in addition to skin complications. Patient 13 developed thigh ischemia and subsequent partial necrosis of his quadriceps and hamstrings as well as necrosis of his skin flap. He underwent debridement of the skin as well as the quadriceps and hamstring muscles, ultimately requiring an additional 15 debridement surgeries with wound VAC changes over a period 2 months after his original rotationplasty before his wounds completely healed. This case was responsible for 40% of the unplanned surgeries performed in this series. Despite the extensive surgical treatment required postoperatively, he ultimately recovered and had an excellent outcome at his most recent follow-up appointment 3.5-years postoperatively with active hip flexion and extension with excellent gait using his prosthesis. 

**Figure 8:**
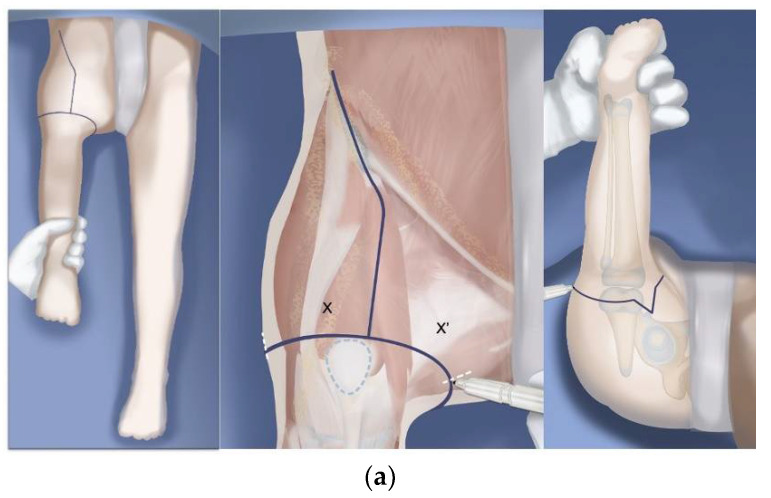

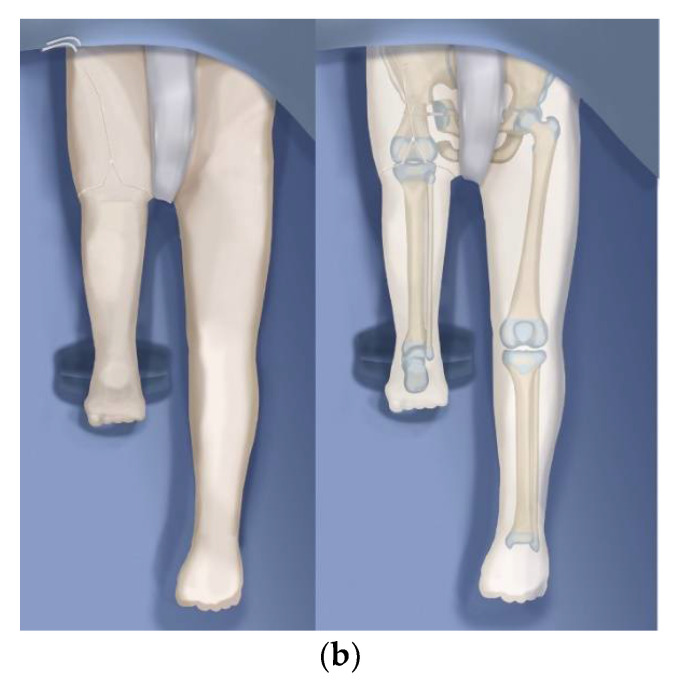
(**a**) Illustration of the new Pinsky incision line for Brown, Paley–Brown and Paley type rotationplasties. The 3cm equilateral triangle on the posteromedial side is located at 5 o’clock for right legs and 7 o’clock for left legs. (**b**) After closure of the Pinsky incision with the triangle inserted anteriorly between the flaps. The wound edges except the triangle are all resected back one cm or more before closure.

As a result of the high rate of wound complications we now use a plastic surgeon to close the incision at the end of the surgery. This has reduced our wound complication rate. Dr. Mark Pinsky the plastic surgeon we use has modified the incision as shown in Figure 8a,b. Prior to closure, he resects one cm of all the wound edges on both sides of the wound. He shortens some parts of the flaps depending on the redundancy of the skin. He also meticulously closes the basal and interposing dead space with a multilayer closure of the wound edges. Since rotationplasty is such a long surgery taking between 6–12 h of surgery prior to the closure, using a plastic surgeon removes the exhausted orthopedic surgeon who did the surgery and replaces him/her with a fresh surgeon who is an expert at soft tissue closure. 

Ischemia complications from arterial or venous obstruction is a dreaded complication that can lead to partial or complete loss of the limb, a rare, but known risk associated with rotationplasty. In the cancer literature ischemic complications in rotationplasty was reported in 5.2–10% of cases with 4.3–6.7% ultimately leading to amputation [14,21,22]. In this study specific to CFD, there was one compartment syndrome of some thigh muscles but no arterial or venous obstruction leading to ischemia. The key to avoiding this problem is to skeletonize the femoral artery and vein so that there are no attached tethering vessels that could kink the femoral artery or vein. All patients in this study also have a preoperative magnetic resonance angiogram to study the circulation and to rule out a dominant ischiadic artery and vein with an atretic femoral artery and vein [23]. We only had one such patient in this study. 

Patient 10 was found to have sciatic nerve injury postoperatively. He was taken back to the OR on postoperative day #3 for exploration and was found to have his sciatic nerve compressed between the femur and the pelvis. The femur was abducted to decompress the nerve. This was a case of Paley type RP and the ischial ramus had not been resected. The hip was being held in fixed neutral position with a temporary spanning plate. After abducting the hip by repositioning the temporary arthrodesis plate, the sciatic nerve fully recovered, and he has no nerve deficit at most recent follow-up appointment 3.5 years postoperatively. Documented causes of sciatic nerve injury in the literature after rotationplasty include kinking of the nerve, compression from hematoma and compression from excessively retained muscle [11,12,22,24]. Compression of the sciatic nerve between the femur and pelvis has not been previously reported and is specific to the medialization of the femoral segment. For this reason, the ischial ramus should be resected in both the Paley–Brown and the Paley types of RP. 

Patient 11 in this series developed a delayed union of a proximal tibial osteotomy that required surgical management. In addition to RP, his original surgery included a rotational osteotomy through the proximal tibia to externally rotate the ankle into better alignment. The tibial osteotomy went on to a delayed union and was treated with compression and bone grafting from the iliac crest at 4-months postoperatively. It healed promptly after this.

Delayed and non-union following rotationplasty are known complications, well documented in rotationplasty for tumor indications. In the cancer literature, delayed and non-union after RP was reported in up to 8.6% of cases [13,14,21]. RP done for tumor indications carry additional risk factors for poor healing including chemotherapy and radiation, that are not typical in CFD patients. In this study in CFD patients, delayed union after RP occurred in 1/19 (5.3%) of patients (proximal tibia osteotomy), which is lower than the published incidence in the literature after RP for tumor patients. No patients developed a delayed or non-union at the junction of the femur with the pelvis. 

Appropriate coronal plane lower extremity alignment after rotationplasty in CFD is particularly important given most techniques fuse the femur to the pelvis, thus eliminating abduction and adduction. Brown [19] specifically described the importance of the distal femoral joint surface being parallel to a line connecting the bottom of the two ischia at the end of the surgery. This can be difficult when fusing the remnant femur to the side of the pelvis in the Brown technique, given it is not an orthogonal surface. The Paley modification of the Brown technique creates a broad orthogonal surface in the Chiari osteotomy that facilitates alignment and healing. In this study, overall coronal pelvo-lower extremity alignment was measured on full radiographs retrospectively at 6 weeks postoperatively and compared to latest available radiograph, an average of 3.3-years later. The final alignment was about 96°. This included the five cases that required additional surgery to realign the lower limb.

Although the Paley modification of the Brown technique is technically easier to achieve orthogonal alignment, given the small sample groups, alignment between the Brown and Paley–Brown techniques could not be compared in this study. Of the five patients that underwent elective osteotomies to change lower extremity coronal alignment, four out of five were the Paley–Brown technique and only one was in a patient having undergone the Paley type of rotationplasty. This highlights how critical achieving orthogonal alignment in the lower extremity is when fusing the femur to the pelvis and also highlights a key advantage of the Paley type rotationplasty when it possible. As it creates a neo-hip joint that preserves hip abduction and adduction in addition to flexion and extension, it allows the neo-hip to compensate for some lower extremity mal-coronal malalignment, which is not possible in the Brown and Paley–Brown techniques.

Patient 1 and 19 developed progressive distal femur valgus in the post-operative course because of asymmetric and incomplete epiphysiodesis of the distal femur at the time of original surgery. Both underwent femur screw removal and hemi-epiphysiodesis with screw placement on the medial side of the distal femur physis. This was successful in patient 19 and epiphysiodesis was subsequently completed by placing a lateral distal femur epiphyseal screw once valgus was fully corrected. In patient 1 the valgus deformity did not correct after a medial hemi-epiphyseal screw was placed, and he underwent eventual complete epiphysiodesis with distal femur varus osteotomy to correct the valgus malalignment. Although this is not a common complication post-operatively, in his original report Brown [19] had one case of progressive varus deformity of the distal femur that required distal femur valgus osteotomy and complete epiphysiodesis one year post-operatively. Fortunately, at final follow-up of patients 1 and 19 were without clinical coronal plane deformity and walking without obvious deformity nor lurch.

Concomitant distal mal-alignment around the ankle is a common in the CFD population, given the known association with fibular hemimelia. The ankle in fibular hemimelia can range from normal (Paley type 1), a dynamic valgus deformity (Paley type 2) to fixed equino-valgus or varus [25,26]. Furthermore, fibular hemimelia also has internal torsion of the foot relative to the knee. This is compensated by external femoral torsion. However, after fusing the femur to the pelvis with the knee fixed in the frontal plane, this compensation uncovering the internal tibial torsion. For this reason, a derotation osteotomy of the distal tibia to correct the internal torsion should also be done to improve alignment and rotation of the foot relative to the knee for ease and improvement of prosthetic fitting. In this series 4 patients underwent supramalleolar osteotomy of the distal tibia to correct internal torsion. Two were performed as part of the index surgery and 2 were performed as an elective procedure in the late-follow-up.

A new technique was developed by the senior author to treat dynamic valgus ankle instability in fibular hemimelia in 2014, called the SHORDT procedure (Shortening Osteotomy Realignment Distal Tibia) [27]. This should be performed at the time of rotationplasty in patients who have dynamic ankle valgus, commonly found in Paley type 2 fibular hemimelia. In this series, five patients underwent the SHORDT procedure (Figure 4e). Four were performed as part of the index surgery and one was performed as an elective procedure in the late-follow-up. The SHORDT procedure should be performed concomitantly with the rotationplasty if dynamic ankle valgus is present. The one patient in this study who had a SHORDT performed in the late follow-up (patient 6) underwent a supramalleolar osteotomy for internal rotation at her index rotationplasty, however this was prior to the development of the SHORDT.

No patient in this study was found to have late derotation of the rotationplasty at final follow-up. Late derotation has been well documented in the CFD rotationplasty literature historically, especially when performed in children under the age of 12 and is a major disadvantage of the Van Nes rotationplasty technique where the derotation is performed through the knee or distally in the tibia [9,28,29]. Brown [19] postulated that late derotation occurred in the Van Nes rotationplasty because it does not address the unstable hip. Rotational correction through the tibia is not stable since the hip is not anchored. Brown specifically designed his rotationplasty more proximally through the femur to address these concerns and reported no late loss of rotation in his study. Paley–Brown and Paley rotationplasty techniques follow these principles in addressing the unstable hip as well as performing the rotation proximally and likewise in this series no late derotation was observed. Performing a rotationplasty in CFD in early childhood is preferred to allow the child to adapt psychologically and physically. The Paley–Brown and Paley rotationplasty techniques offer the advantage of being able to perform the reconstruction in early childhood (as early as age 3 years).

All seven patients, who had a combined 8 temporary arthrodesis implants placed around the hip or knee at the index procedure, required planned implant removal. Temporary arthrodesis is a technique developed by the senior author (D.P.) in which a joint is temporarily spanned with plate, rod or external fixator in order hold a joint contracture in the corrected position to maintain correction or to augment fixation for healing of an osteotomy [19,30,31,32,33] or to prevent development of contracture during lengthenings. This is particularly useful in the Paley-type rotationplasty as there is little room in the remnant femoral head to achieve solid fixation to the remnant femur with screws alone. The significant forces across the hip joint coupled with a small area for fixation puts the construct at high risk for failure of fixation or nonunion. A temporarily arthrodesis of the hip with hip spanning internal plate from the ilium to the remnant femur neutralizes all forces across the hip joint allowing the femur to fuse to the remnant femoral head. Early in the series the temporary hip arthrodesis was performed with an external fixator that extended to the ipsilateral pelvis and contralateral pelvis (patients 3, 4 5, 7) in both Paley–Brown and Paley, but was ultimately found to be unnecessary in most Paley–Brown type. This was performed with internal plate fixation later in the series as an all-internal temporary hip arthrodesis in all subsequent Paley type (patient 13, 14, 17) and is an important part of the Paley rotationplasty technique. All of these plates were removed routinely at 4-months postoperative.

There are several limitations to this study. First, this study only looked at complications, radiographic alignment and need for secondary surgery in rotationplasty for CFD and did not measure clinical outcomes using a gait lab or questionnaire, etc. It did however report the gait of patients recorded in the chart at final follow-up. All patients were walking well and actively using their prosthetic. No patient was having pain. All patients or parents were satisfied with the outcome of their child and no patient was seeking an alternative to the treatment provided. As noted, there were several late complications addressed to improve prosthetic fitting and gait (supramalleolar osteotomy, SHORDT, proximal femoral realignment osteotomy and advancement of hip extensor muscles). All of these procedures achieved their goals and all patients achieved improved fitting and gait after these secondary elective procedures. There are lessons to be learned from these unplanned late secondary procedures. For example, an intraoperative assessment of the tibial valgus and torsion should be done after completing the hip procedure especially in the Paley–Brown where the knee is solidly fixed to the pelvis. The supramalleolar or SHORDT should be done with the index procedure if the rotation or alignment is not correct. Another example is the hip extensor weakness. This can be prevented by careful transfer and advancement of the gluteus maximus and hamstrings to create strong extension of the hip.

Second, this is a retrospective study. Since this is a rare treatment for a rare condition, 19 cases treated over a 10-year period, almost two per year, a prospective study will take a very long time. As the next step a long-term follow-up outcome questionnaire of this group with gait analysis will reveal additional useful information.

Despite these limitations, this study is the largest study on rotationplasty for CFD. This study also differs from previous reports in the age at time of rotationplasty. The methods discussed can be applied as early as age 2 years, all through childhood into teen age years and even in adulthood (oldest patient was 36 years). This study also presents a variety of rotationplasties dispelling the idea that rotationplasty is a single type of operation. The senior author has modified or developed new rotationplasty techniques to apply to the wide spectrum of deficiency and deformity seen with CFD. While the original Van Nes concept is valid converting the ankle into an actively functioning knee, the instability at the hip makes it less reliable for use in Paley types, 2 and 3 CFD where there is no stability of the proximal femur and hip joint. The Van Nes is therefore most applicable to the Paley type 1a_3_ and 1b cases in which there is an intact femoral head-neck-greater trochanter-shaft unit. Even in these milder CFD types, the original Van Nes does not address the proximal femoral deformity limiting the function of the hip after fusion of the knee and rotationplasty and leaving it prone to degenerative changes at the hip. The Paley modification of combining the SUPERhip procedure in these cases with the Van Nes rotationplasty including knee fusion results in an anatomically normal hip combined with a stable fused knee and a functional ankle which serves as the new knee (Figure 6a–g). Another modification of the Paley–Van Nes is for patients with CFD associated with a congenital knee fusion (Figure 7a–e). In these patients the knee fusion has a flexion deformity associated with a flexion, external rotation deformity at the hip. These patients are not candidates for the Paley–Brown since they have no knee joint. They need stabilization of the upper femur which is only connected to the pelvis with the strong fibrous anlage of the femoral neck. The rotationplasty can be done through the congenital knee fusion but the upper femur also needs release of its flexion deformity and stabilization with a soft tissue sling to prevent the proximal end of the femur from pointing posteriorly. This is achieved by creating a “femoral sling” which eliminates the flexion deformity of the femoral remnant while preserving the fibrous femoral anlage to keep the axial stability of the hip (Figure 7b). The femoral sling is created by wrapping a band of fascia lata originating at the anterior superior iliac spine (ASIS) around the proximal femur and back to the ASIS. Therefore, the PaleySUPERhip–Van Nes and the PaleySling–Van Nes have specific indications depending on the type of femoral deficiency and ways of stabilizing the upper femur. 

Similarly, the Brown (Figure 2a–f) and Paley–Brown (Figure 3a–d) rotationplasties are specifically indicated for specific CFD deficiencies. They are best suited to Paley type 2b, or 3a and 3b cases with a functioning knee joint (Figure 2a). Even in Paley type 3b cases where the knee has limited motion, the motion of the knee can be increased by the posterior knee capsulotomy which is part of the procedure. As long as the knee can act as a hinge joint for flexion-extension, fusion of the distal femur remnant to the pelvis will give a stable uni-directional functioning hip joint. The difference between the Paley–Brown and the Brown is the location of the femur, which is placed much more medial in the Paley–Brown. The choice to do a Brown instead of a Paley–Brown, in one case in this series (Figure 2c–f), was indicated because the patient was obese making the medialization of the Paley–Brown difficult. The femur was fixed to the side of the pelvis facilitating the closure and soft tissue reconstruction. 

The Paley rotationplasty is indicated in Paley type 2a and 3a or b where there is a mobile femoral head in the acetabulum (Figure 4a–e). The determination of a mobile femoral head is made from the preoperative MRI and from intraoperative capsulotomy and direct examination. The coronal cuts of the MRI will show a patent femoral head-acetabulum joint. The axial cuts will show whether this femoral head remains round in cross section and whether there is a synostosis or synchondrosis between the posterior aspect of the femoral head and the ischium. In cases that this synostosis or synchondrosis is present the femoral head is considered fused to the acetabulum and is not a candidate for the Paley type and should be treated instead by the Paley–Brown rotationplasty. The theoretical advantage of the Paley type over the Paley–Brown is that the former preserves hip abduction-adduction which is not possible in the latter. Whether this gives better or worse gait will require gait studies to determine. Both groups walked well in the subjective assessment done at follow-up visits.

When there is no knee joint with no or very little residual femur (Paley type 3c) (Figure 5a) the hip can be stabilized by inserting the cartilage covered lateral side of the tibial plateau or residual femur condyle into the acetabulum. This assumes there is a non-mobile femoral head present that can be enucleated (Paley–Winkelmann rotationplasty) (Figure 5b). To allow this to scar into place and not redislocate a suture anchor or a syndesmotic suture-double washer is used to allow flexion extension motion of the hip, while keeping the new femoral head in place (Figure 5c). These cases are the rarest type to present and therefore the least common rotationplasty to consider. The Winkelmann approach, however, allows the surgeon to recreate hip stability and permit direct weight transfer in an axial fashion between the tibia and the pelvis. This direct weight transfer is preferable and more stable than the floating hip of the original Van Nes or even of the PaleySling–Van Nes.

## 5. Conclusions

In summary, indication specific rotationplasty remains a very successful method of addressing the severe degree of femoral deficiency, deformity and discrepancy in patients with CFD, despite high rates of wound complications. The information learned from this preliminary study will be helpful to improve the results of the next cohort of patients treated by rotationplasty. 

## Figures and Tables

**Table 1:** Patient Characteristics.

Patient	Age at Surgery (Years)	Follow-Up (Years)	CFD ^1^ PALEY CLASSIFICATION	SIDE	Rotationplasty Type	Concomitant Procedures
1	2.0	9.6	Paley Type 3b	Right	Paley–Brown	None
2	2.1	7.5	Paley Type 3b	Right	Paley–Brown	None
3	10.0	7.0	Paley Type 3b	Right	Paley–Brown	1. Hip temporary arthrodesis (exfix)
4	14.2	6.8	Paley Type 3b/ MHE ^2^	Right	Brown	1. Hip temporary arthrodesis (exfix)
5	2.0	6.7	Paley Type 3a	Right	Paley	1. Knee temporary arthrodesis (rod)
						2. Hip temporary arthrodesis (exfix)
6	6.4	6.7	Paley Type 3b	Right	Paley–Brown	1. Supramalleolar tibial osteotomy
7	2.9	1.8	Paley Type 3a	Left	Paley	1. Hip temporary arthrodesis (exfix)
8	12.6	1.8	Paley Type 3c	Left	Paley–Winkelmann	None
9	4.4	1.8	Paley Type 2b	Right	Paley–Brown	None
10	10.2	2.0	Paley Type 3b	Left	Paley–Brown	None
11	3.9	1.6	Paley Type 3c	Right	Paley–Winkelmann	1. Proximal tibial derotational osteotomy
12	36.9	0.8	Paley Type 1b	Right	Paley–Van Nes	1. SUPERHip
						2. Supramalleolar tibial osteotomy
13	12.4	2.5	Paley Type 2b	Right	Paley	1. Hip temporary arthrodesis (plate)
14	5.2	1.5	Paley Type 3b	Right	Paley	1. SHORDT ^3^ procedure
						2. Hip temporary arthrodesis (plate)
15	8.4	0.7	Paley Type 3a	Left	Paley–Brown	1. SHORDT procedure
16	10.9	1.2	Paley Type 2c	Right	Paley–Brown	None
17	9.4	0.9	Paley Type 3a	Left	Paley	1. SHORDT procedure
						2. Hip temporary arthrodesis (plate)
18	3.9	0.6	Paley Type 3b	Right	Paley–Brown	None
19	4.7	0.5	Paley Type 3a	Left	Paley–Brown	1. SHORDT procedure

^1^ CFD indicates congenital femoral deficiency. ^2^ MHE indicates multiple hereditary exostosis. ^3^ SHORDT indicates shortening osteotomy realignment distal tibia.

**Table 2:** Complications.

Patient	Complication	Additional Surgery
1	1. Progressive valgus distal femur, failed epiphysiodesis	Distal femur medial hemi-epiphysiodesis
	2. Valgus distal femur, failed hemi-epiphysiodesis	Distal femur varus osteotomy, repeat epiphysiodesis
2	None	None
3	None	None
4	1. Wound necrosis/dehiscence	Debridement and secondary closure x3
5	1. Pin site infection	None
6	None	None
7	1. Wound necrosis/dehiscence	None
	2. Pin site infection	None
8	None	None
9	None	None
10	1. Wound necrosis/dehiscence	Debridement and secondary closure x1
	2. Right sciatic nerve palsy/entrapment	Abducted femur to decompress sciatic nerve
11	1. Wound necrosis/dehiscence	Debridement and secondary closure x3
	2. Tibial delayed union	Compression with iliac crest bone graft
12	None	None
13	1. Wound necrosis/dehiscence	Debridement and secondary closure x15
	2. Quadricep and hamstring compartment syndrome	
14	1. Wound necrosis/dehiscence	Debridement and secondary closure x4
15	1. Wound necrosis/dehiscence	Debridement and secondary closure x1
16	1. Wound necrosis/dehiscence	Debridement and secondary closure x2
17	1. Wound necrosis/dehiscence	Debridement and secondary closure x1
18	None	None
19	1. Wound necrosis/dehiscence	Debridement and secondary closure x1
	2. Progressive valgus distal femur, failed epiphysiodesis	Distal femur medial hemi-epiphysiodesis
		Distal femur repeat epiphysiodesis

**Table 3:** Index and Follow-up Osteotomies.

Patient	Osteotomy of the Distal Tibia	Osteotomy of the Femur for Alignment	Osteotomy of the Upper Tibia for Alignment
1	None	1. Distal femur varus osteotomy @ follow-up	None
2	None	1. Distal femur varus osteotomy @ follow-up	None
3	None	None	1. Proximal tibia valgus osteotomy @ follow-up
4	1. Supramalleolar osteotomy @ follow-up	None	None
5	1. Supramalleolar osteotomy @ follow-up	None	None
6	1. Supramalleolar osteotomy @ index surgery	1. Distal femur varus osteotomy @ follow-up	None
	2. SHORDT ^1^ procedure @ follow-up		
7	None	1. Distal femur valgus osteotomy @ follow-up	None
8	None	None	None
9	None	None	None
10	None	None	None
11	None	None	1. Proximal tibia derotational osteotomy @ index surgery
12	1. Supramalleolar osteotomy @ index surgery	None	None
13	None	None	None
14	1. SHORDT procedure @ index surgery	None	None
15	1. SHORDT procedure @ index surgery	None	None
16	None	None	None
17	1. SHORDT procedure @ index surgery	None	None
18	None	None	None
19	1. SHORDT procedure @ index surgery	None	None

^1^ SHORDT indicates shortening osteotomy realignment distal tibia.

## Data Availability

The data presented in this study are available on request from the corresponding author. The data are not publicly available due to patient privacy concerns.
